# Lytic potential of *Lysobacter capsici* VKM B-2533^T^: bacteriolytic enzymes and outer membrane vesicles

**DOI:** 10.1038/s41598-020-67122-2

**Published:** 2020-06-19

**Authors:** A. S. Afoshin, I. V. Kudryakova, A. O. Borovikova, N. E. Suzina, I. Yu. Toropygin, N. A. Shishkova, N. V. Vasilyeva

**Affiliations:** 1 Laboratory of Microbial Cell Surface Biochemistry, G.K. Skryabin Institute of Biochemistry and Physiology of Microorganisms, Russian Academy of Sciences, PSCBR RAS, 5 Prosp. Nauki, Pushchino, Moscow Region 142290 Russia; 2 Department of Proteomics, V.N. Orekhovich Research Institute of Biomedical Chemistry, Russian Academy of Medical Sciences, 10 Pogodinskaja Str., Moscow, 119832 Russia; 3 Laboratory of Anthrax Microbiology, FBIS State Research Center for Applied Microbiology and Biotechnology, Obolensk, Serpukhov District, Moscow Region 142279 Russia

**Keywords:** Secretion, Antimicrobials, Applied microbiology, Membranes

## Abstract

Recent recurrent outbreaks of bacterial resistance to antibiotics have shown the critical need to identify new lytic agents to combat them. The species *Lysobacter capsici* VKM B-2533^T^ possesses a potent antimicrobial action against a number of bacteria, fungi and yeasts. Its activity can be due to the impact of bacteriolytic enzymes, antibiotics and peptides. This work isolated four homogeneous bacteriolytic enzymes and a mixture of two proteins, which also had a bacteriolytic activity. The isolates included proteins identical to *L. enzymogenes* α- and β-lytic proteases and lysine-specific protease. The proteases of 26 kDa and 29 kDa and a protein identified as N-acetylglycosaminidase had not been isolated in *Lysobacter* earlier. The isolated β-lytic protease digested live methicillin-resistant staphylococcal cells with high efficiency (minimal inhibitory concentration, 2.85 μg/mL). This property makes the enzyme deserving special attention. A recombinant β-lytic protease was produced. The antimicrobial potential of the bacterium was contributed to by outer membrane vesicles (OMVs). *L. capsici* cells were found to form a group of OMVs responsible for antifungal activity. The data are indicative of a significant antimicrobial potential of this bacterium that requires thorough research.

## Introduction

Microorganisms producing antimicrobial substances have always been of heightened interest for the scientific community. This is due to the search for novel antimicrobial compounds to control multiple drug resistant pathogens, research into features of their synthesis by the microbial cell and understanding of microbe–microbe interactions under the ambient conditions. Some microorganisms stand out for their ability to produce complexes of antimicrobial compounds that consist of various hydrolytic, including bacteriolytic, enzymes, as well as of antibiotics and antimicrobial peptides. These complexes possess potent lytic activities owing to synergic effects of their antimicrobial constituents. Producers of lytic complexes have tremendous advantages and are highly competitive under conditions of the environment. The hunt for such microorganisms is very active nowadays as they are the source of novel antimicrobial agents for medicine, veterinary and agriculture.

Representatives of the genus *Lysobacter* are some of the highly active producers of lytic agents. The genus was formed in 1978 by the Canadian microbiologists^1^. At present, the genus amounts to over 40 species (http://www.bacterio.net/lysobacter.html). Accessible in the databases are the complete genomes (assembly level according to the NCBI) of 11 strains (*Lysobacter* sp. SJ-36, *Lysobacter* sp. TY2–98, *L. capsici* 55, *L. capsici* KNU-14, *L. antibioticus* ATCC 29479, *L. antibioticus* 76, *L. maris* HZ9B, *L. lycopersici* CC-Bw-6, *L. enzymogenes* YC36, *L. enzymogenes* M497-1, *L. gummosus* 3.2.11) (https://www.ncbi.nlm.nih.gov/genome/?term=lysobacter). Analysis of the genomes indicates the occurrence of genes responsible for the synthesis of bacteriolytic enzymes, for the biosynthetic pathways of antibiotics and antimicrobial peptides^2,3^. The following have been isolated and studied to date in greatest detail: bacteriolytic enzymes in *Lysobacter* sp. XL1^4–8^; antibiotics and antimicrobial peptides in *L. enzymogenes*^9–12^, *L. albus*^13^, *L. antibioticus*^14–16^, *L. lactamgenus*^17,18^, *L. gummosus*^19^, *Lysobacter* sp. RH2180-5^20^, *Lysobacter* sp. ATCC 53042^21^, *Lysobacter* sp. BMK333-48F3^22^, *Lysobacter* sp. SB-K88^23^.

The antimicrobial potential is significantly contributed to by outer membrane vesicles (OMVs) formed by bacterial cells of this genus. The ability to form OMVs has been first shown for *Lysobacter* sp. XL1^8^. Outer membrane vesicles of this bacterium contain the bacteriolytic protease L5 and have a potent bacteriolytic and curative action with respect to model infections^24^. To date, it has been shown that OMVs of *L. enzymogenes* C3 may contain a polycyclic tetramate macrolactam (dihydromaltophilin, alteramide B)^25^.

Noteworthy among *Lysobacter* bacteria is *L. capsici*. Some strains of this species have been relatively investigated. Genomic studies of strains *L. capsici* 55*, L. capsici* KNU-14, *L. capsici* AZ78, *L. capsici* X2–3 have been carried out^2,26,27^, https://www.ncbi.nlm.nih.gov/genome/?term=lysobacter). The transcriptome of *L. capsici* AZ78 has been studied at the interaction with target cells of phytopathogenic fungi^28^. A potent antifungal action of *L. capsici* AZ78^29,30^ and *L. capsici* PG4^31^ has been shown. The anti-nematode agents chitinase, gelatinase and lactic acid have been isolated from *L. capsici* YS1215 and characterized^32–34^. An anti-oomycete cyclic dipeptide cyclo(L-Pro-L-Tyr) has been isolated from *L. capsici* AZ78 and characterized^29^. Genomic studies are also indicative of the occurrence of bacteriolytic enzymes. However, this has not been confirmed earlier experimentally.

Analysis of the literature suggests a potent lytic potential of this species. Still, the lytic properties of the type strain *L. capsici* YC5194^Т^ = VKM B-2533^Т^ have not been investigated earlier. The aim of this work was to study the lytic potential of the bacterium *L. capsici* VKM В-2533^Т^ (dependence of the production of lytic agents on storage and cultivation conditions; production of bacteriolytic enzymes and outer membrane vesicles).

## Results

### Dependence of the production of lytic agents in *L. capsici* VKM B-2533^T^ on storage and cultivation conditions

The issue of stable production of commercially valuable substances by microorganisms has always been topical. In *L. capsici*, the dependence of the total production of antimicrobial agents on storage and cultivation conditions has not been investigated earlier.

Upon acquisition of the *L. capsici* type strain from the All-Russian Collection of Microorganisms RAS, we investigated its ability to lyse live cells of various microorganisms. The strain was found to have a potent lytic action against Gram-positive bacteria, yeasts and fungi. No activity was revealed with respect to Gram-negative bacteria. In the laboratory collection the strain was maintained on agarized LBs and PMA media and in cryopreservation. The culture stored on agarized media was re-inoculated each ten days. A smaller number of passages led to a viability loss.

After a year under the selected storage conditions a 100% antimicrobial activity was preserved only in the cryopreserved culture. In this case, the activity was revealed in cultivation on all tested liquid nutrient media.

**Figure 1:**
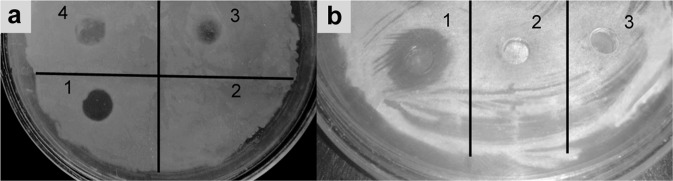
Lytic action of the culture liquid of *L. capsici* grown on SYM (1), LB (2), KSP (3) and RM (4) media with respect to live test objects: (**а**) *B. cereus*; (**b**) *F. solani*.

In storage on LBs medium, the antimicrobial activity was lost partially. After a year of maintenance on this medium, the activity with respect to all bacterial test objects was revealed on SYM, KSP and RM media (Table 1, Fig. 1a). It is seen from the table that at OD/mL and LU/mL values of the same order of magnitude on different cultivation media the lytic potential with respect to live test objects is not the same. Thus, during the cultivation of the bacterium on PYKM medium the lytic action with respect to live test cultures is totally absent. Herewith, the values of OD/mL and LU/mL on this medium are the same as on SYM and KSP. At high values of LU/mL during the cultivation on RM and LB media the lytic action with respect to live test objects is observed only on RM medium. The most potent lytic activity with respect to live test cultures was observed on SYM medium. The yeast-lytic activity was preserved with respect to *C. boidinii*, being revealed in cultivation on virtually all media. With respect to *C. utilis* the activity was lost. The antifungal activity was revealed in cultivation on only SYM media (Table 1, Fig. 1b). In storage on PMA medium and subsequent cultivation on various liquid nutrient media, the antimicrobial activity was gradually lost. After a year of this storage, a weak lytic activity was revealed against bacteria *M. luteus*, *M. reseus*, *S. aureus* at the cultivation on RM medium and with respect to *M. luteus* in cultivation on KSP medium (Table 1). The yeast-lytic activity was preserved in the same way as during the maintenance of the culture on LBs. The antifungal activity was totally lost.

**Table 1:** Dependences of the production of lytic agents in *L. capsici* VKM B-2533^T^.

Storage medium	LBs						PMA					
Growth medium	5/5	PYKM	SYM	KSP	RM	LB	5/5	PYKM	SYM	KSP	RM	LB
OD/mL	4.2 ± 0.6	4.5 ± 1.5	3.95 ± 0.47	3.11 ± 0.27	4.9 ± 0.2	5.8 ± 1.3	6.2 ± 1.6	6.8 ± 0.3	3.4 ± 1.4	3.7 ± 0.1	5.7 ± 0.4	7.2 ± 0.8
LU/mL	50.0 ± 8.5	168.4 ± 36.8	174.0 ± 18.7	174.4 ± 36.2	354.8 ± 32.2	338.2 ± 186.4	37.2 ± 8.5	270.8 ± 47.0	13.6 ± 5.7	171.7 ± 15.1	542.6 ± 16.1	155.2 ± 52.0
**Bacteria**												
*Micrococcus luteus*	–	–	++	++	+	–	–	–	–	+	+	–
*Bacillus cereus*	–	–	++	+	+	–	–	–	–	–	–	–
*Micrococcus roseus*	–	–	++	+	+	–	–	–	–	–	+	–
*Staphylococcus aureus*	–	–	++	+	+	–	–	–	–	–	+	–
**Fungi and yeasts**												
*Aspergillus niger*	–	–	+	–	–	–	–	–	–	–	–	–
*Fusarium solani*	–	–	+	–	–	–	–	–	–	–	–	–
*Sclerotinia sclerotiorum*	–	–	+	–	–	–	–	–	–	–	–	–
*Candida boidinii*	–	+	++	++	++	+	–	++	++	++	++	+
*Candida utilis*	–	–	–	–	–	–	–	–	–	–	–	–

on storage and cultivation conditions ++, strong lytic effect; +, medium lytic effect; –, no lytic effect.

It should be understood that the total lytic activity can be contributed to by bacteriolytic enzymes, peptides and antibiotics, as well as by OMVs that contain these substances. A crucial condition for the antimicrobial properties of *L. capsici* VKM B-2533 to be retained is its maintenance in cryopreserved state. In this case, the activity is preserved and manifests itself at the cultivation of the bacterium on various nutrient media. The culture maintained on agarized media partially or totally loses its specific activities, which is indicative of a gradual switching-off of individual genes or genes of the whole biosynthetic pathways of some or other agents. However, this feature can be useful in future research into the regulation of the synthesis of these compounds, as well as can facilitate the isolation of specific agents.

### Isolation of bacteriolytic enzymes

**Figure 2:**
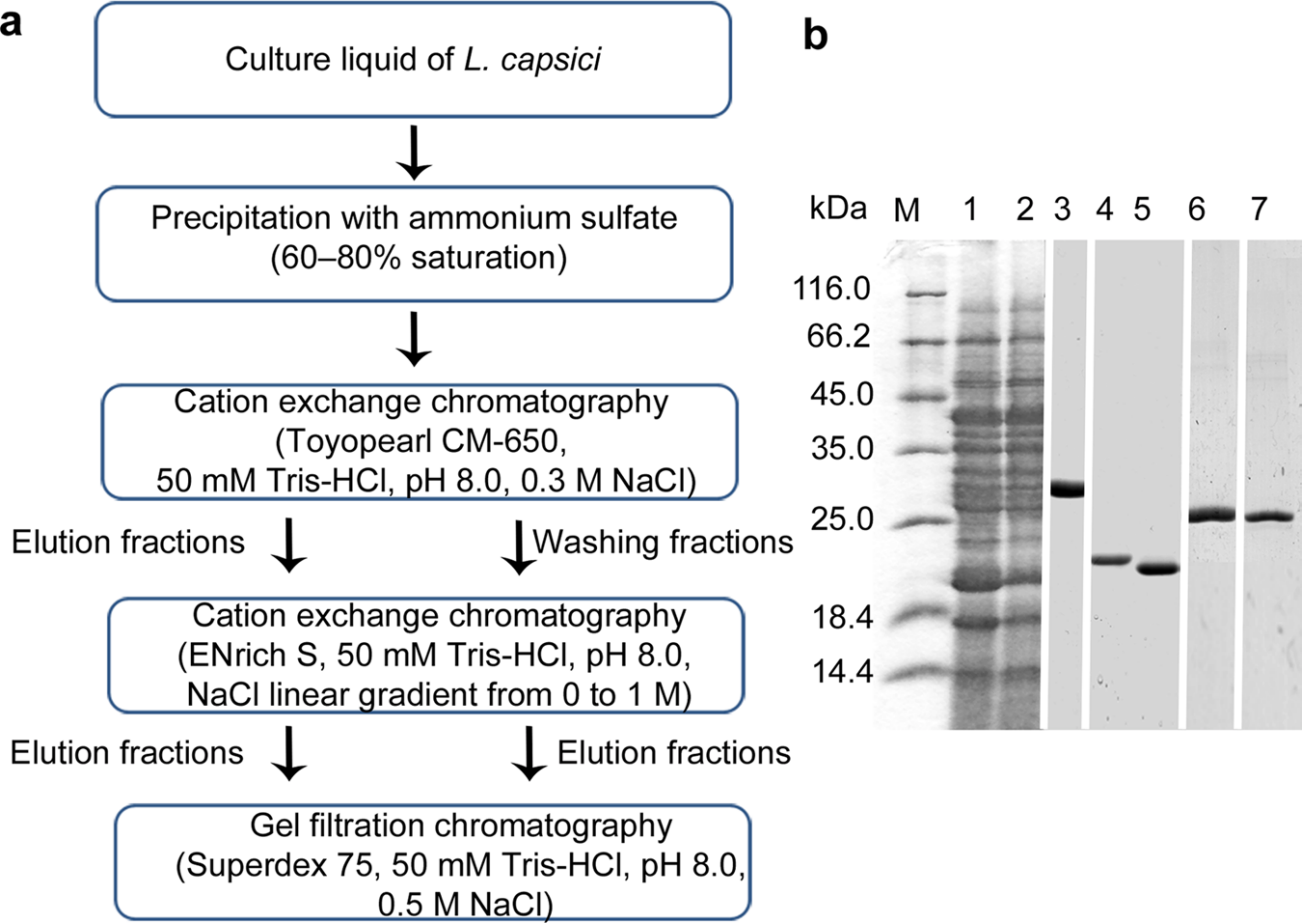
Production of *L. capsici* bacteriolytic enzymes. (**a**) Scheme of purification of bacteriolytic proteins. (**b**) SDS-PAGE: 1, *L. capsici* culture liquid (Supplementary Fig. S1)*;* 2, wash-out after Toyopearl CM-650 (Supplementary Fig. S1); 3, mixture of two serine trypsin-like proteases (Supplementary Fig. S2); 4, α-lytic protease (Supplementary Fig. S2); 5, β-lytic protease (Supplementary Fig. S2); 6, N-acetylglucosaminidase (Supplementary Fig. S3); 7, serine protease (Supplementary Fig. S3).

It is known that extracellular bacteriolytic enzymes are mainly alkaline proteins whose molecular weights, as a rule, do not exceed 30 kDa^8,35,36^. For isolation of *L. capsici* bacteriolytic enzymes, we developed a scheme, which includes fractionation of proteins by ammonium sulphate, cation exchange chromatography and gel filtration (Fig. 2a, Supplementary Table S1). As the result of the developed purification scheme, we succeeded in isolating a mixture of two proteins with MW 29 kDa (Fig. 2b, lane 3) with the total activity 7 367 LU/mg and homogeneous proteins of 21 kDa and 19 kDa (Fig. 2b, lanes 4 and 5) with the activities 13 000 LU/mg and 30 345 LU/mg, respectively. From the wash-out after the first cation exchange chromatography (Fig. 2b, lane 2), in the course of the further purification we isolated two electrophoretically homogeneous proteins of MW 26 kDa (Fig. 2b, lanes 6 and 7) with the activities 55 LU/mg and 598 LU/mg, respectively.

Using MALDI-TOF, we found not only the known and earlier characterized proteins among those isolated but also absolutely new proteases not isolated earlier (Table 2). The mixture of proteins was found to consist of two serine proteases. One of them was identified for the first time. Proteins of МW 21 and 19 kDa are the α- and β-lytic proteases well known for *Lysobacter*^37^. Protein identified as N-acetylglucosaminidase, and one more serine protease of MW 26 kDa, were isolated in *Lysobacter* for the first time.

**Table 2:** Bacteriolytic enzymes of *L. capsici* VKM B-2533^T^ identified by MALDI-TOF.

Proteins identified by MALDI-TOF	Score with protein annotated in NCBI database	MW, kDa	Identity with earlier isolated proteins in *Lysobacter*
**Protein mixture**			
Serine trypsin-like protease	102 with ALN85997.1	29	77.26% with lysine-specific serine protease (P15636.1) *L. enzymogenes*^70^.
Serine trypsin-like protease*	120 with ALN88394.1	29	28% with lysine-specific serine protease (P15636.1) *L. enzymogenes*^70^.
**Homogeneous proteins**			
α-Lytic protease	103 with WP_046657152.1	21	77.83% *L. enzymogenes* (P00778.3)^71^.
β-Lytic protease	98 with ALN84242.1	19	62% *L. enzymogenes* (P27458.1)^72^.
N-acetylglucosaminidase*	306 with KWS04732.1	26	NA**
Serine protease*	207 with WP_096414111.1	26	28.64% with lysine-specific serine protease (P15636.1) *L. enzymogenes*^70^.

* new isolated proteins of the genus Lysobacter. ** not applicable.

Although all isolated proteins possessed bacteriolytic activities with respect to autoclaved staphylococcal cells, only the β-lytic protease (EC 3.4.24.32) possessed a lytic activity with respect to live cells of *S. aureus* 55 MRSA. The minimal inhibitory concentration **(**MIC) of this protein was 2.85 μg/mL. This property determines a high commercial value of this protein.

### Production of *L. capsici* VKM B-2533^T^ recombinant β-lytic protease

The β-lytic protease of *L. enzymogenes* is known since the 1960s;^37^ no recombinant protein has been produced until now, however.

The complete nucleotide sequence of the gene *βl* of the *L. capsici* VKM B-2533^T^ β-lytic protease was determined and entered into the GenBank under the number MN604699.

**Figure 3:**
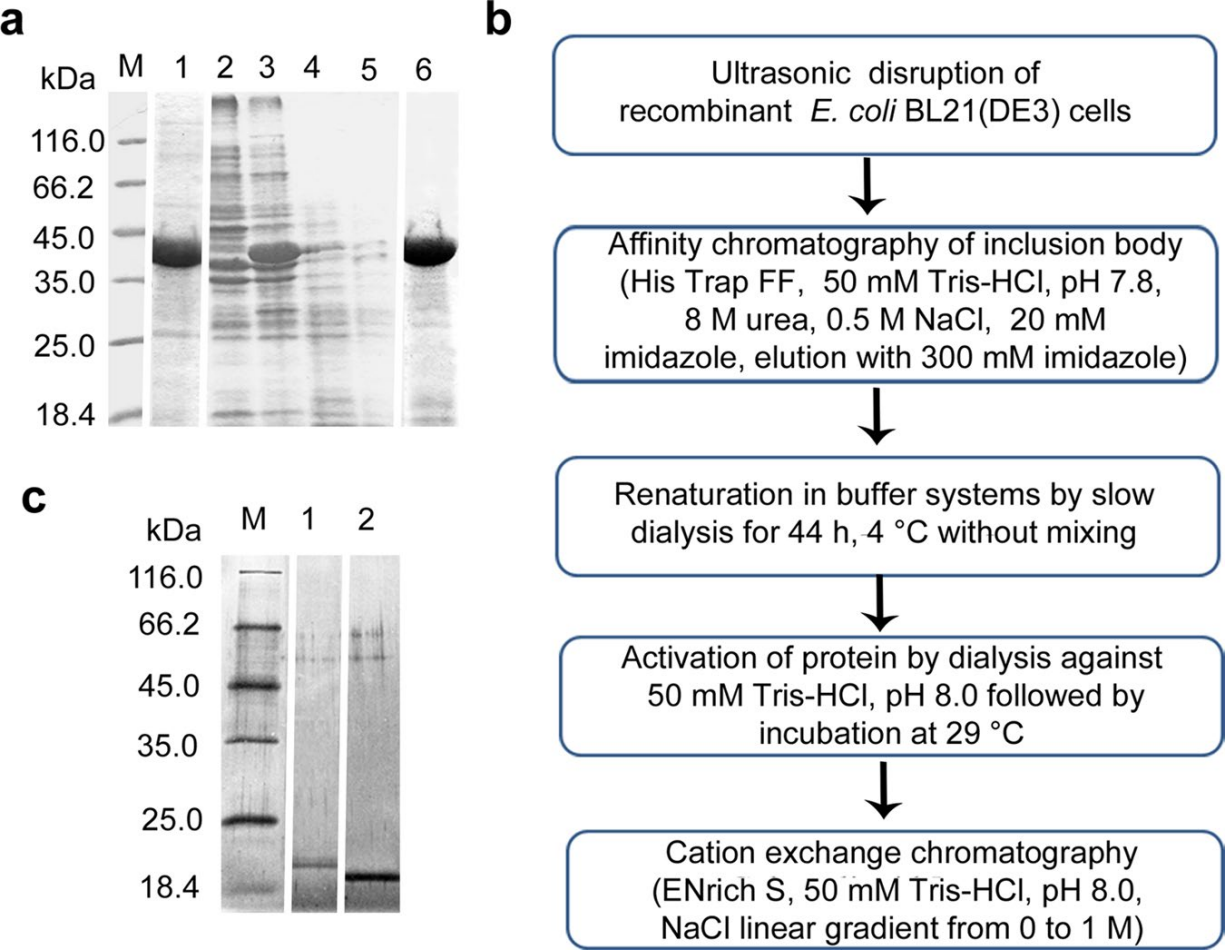
Production of recombinant β-lytic protease. (**a**) SDS-PAGE: 1, inclusion bodies (Supplementary Fig. S4); 2, 4, residue and supernatant, respectively, after the disruption of the *E. coli* BL21(DE3)/pLysE control sample (Supplementary Fig. S5); 3, 5, residue and supernatant, respectively, after the disruption of induced *E. coli* BL21(DE3)/pLysE cells (Supplementary Fig. S5); 6, β-lytic protease purified from inclusion bodies (Supplementary Fig. S4). The gel was stained with a solution of Coomassie Brilliant Blue R-250. (**b**) Scheme of the production of the active form of recombinant β-lytic protease from inclusion bodies. (**c**) SDS-PAGE: 1, recombinant β-lytic protease after renaturation and purification using cation exchange chromatography (Supplementary Fig. S6); 2, β-lytic protease produced from an *L. capsici* producing strain (Supplementary Fig. S6). The gel was stained with imidasole–ZnCl_2_ solutions.

The amplified gene of the *L. capsici* β-lytic protease (1059 bp) was successfully cloned into the expression vector pET19mod, followed by the transformation into cells of *E. coli* BL21(DE3)/pLysE. As the result of this gene expression, cells of the recombinant strains accumulated the major protein of MW 38 kDa (Fig. 3a, lane 1). This corresponds to the weight of the β-lytic protease pro-protein, calculated in the ExPaSy bioinformatics programme (https://web.expasy.org/compute_pi/). Ultrasonic disruption of cells followed by centrifugation produced a residue and a supernatant, which were analyzed electrophoretically. Cells of *E. coli* BL21(DE3)/pLysE, which do not induce IPTG, were used as a control. As seen in Fig. 3a, the protein is localized in inclusion bodies (Fig. 3a, lane 3) and is absent in the supernatant (Fig. 3a, lane 5). The use of such approaches as cultivation at low temperature (20 °C), induction by a reduced concentration of IPTG (0.2 mM) as well as the use of the plasmid pT-GroE carrying the gene of the chaperone GroEL failed to transfer the protein from inclusion bodies to a soluble form. Further work was conducted with inclusion bodies.

The recombinant protein was produced according to the developed scheme (Fig. 3b). Special conditions were selected for renaturation of the β-lytic protease. For this, a number of buffers were used (see Materials and methods). The refolding was considered to be successful if a bacteriolytic activity with respect to live and autoclaved *S. aureus* 209 P cells emerged. The successful refolding was found to occur when using buffers 2, 4, 5 and conditions specified in Fig. 3b.

The recombinant protein was purified to an electrophoretically homogeneous state (Fig. 3c, lane 1). The specific activity on autoclaved and live *S. aureus* 209 P cells was 4 400 LU/mg. The specific activity of the native β-lytic protease with respect to these substrates was 45 000 LU/mg. Although the recombinant protein was an order of magnitude less active, the produced expression system can be considered successful and applicable for scientific purposes.

### Lytic potential of *L. capsici* VKM B-2533^T^ outer membrane vesicles

The subject matter of outer membrane vesicles has been intensively developed in recent years. It is clear even now that OMVs play an important role in the activities of Gram-negative bacteria. Strain *L. capsici* VKM B-2533^T^ possesses a potent lytic potential, and OMVs can be suggested to make a contribution.

**Figure 4:**
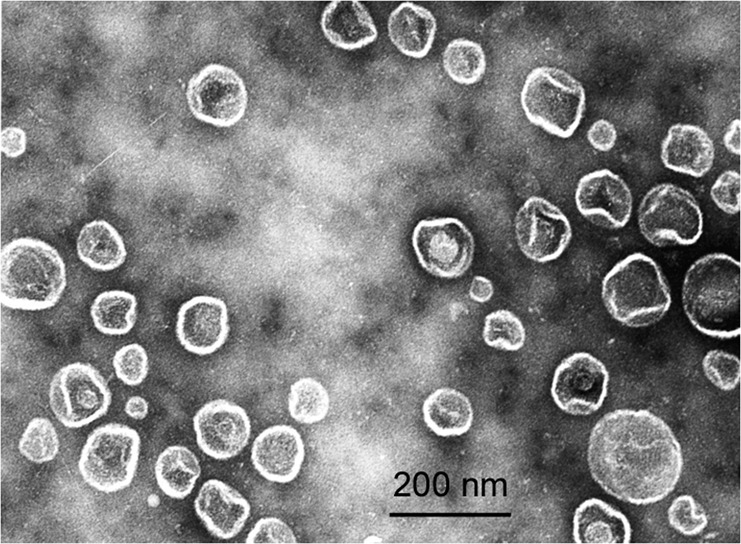
Negative staining electron microscopy of a preparation of *L. capsici* outer membrane vesicles.

Differential centrifugation of the *L. capsici* culture liquid yielded a residue, which was analyzed by electron microscopy. The residue was found to consist of whole OMVs 50 up to 200 nm in diameter (Fig. 4).

The lytic potential of vesicles was studied with respect to bacteria, fungi and yeasts (Table 3, Supplementary Fig. S7). Outer membrane vesicles were found to possess a potent antimicrobial action against all chosen test cultures. Interestingly, the antifungal activity was totally associated with OMVs and disappeared in the surfactant after their precipitation from the culture liquid (Supplementary Fig. S7a). At the same time, an antibacterial activity was revealed both in OMVs and in the culture liquid after their precipitation (Supplementary Fig. S7b).

**Table 3:** Lytic action of OMVs produced by *L. capsici* VKM B-2533^T^.

Microorganisms	Culture liquid before isolation of OMVs	Culture liquid after isolation of OMVs	OMVs
**Bacteria**			
*Micrococcus roseus*	++	++	++
*Staphylococcus aureus*	++	++	++
*Micrococcus luteus*	++	++	++
*Bacillus cereus*	++	+	++
**Fungi and yeasts**			
*Sclerotinia sclerotiorum*	++	–	++
*Fusarium solani*	++	–	++
*Aspergillus niger*	++	–	++
*Candida utilis*	++	–	++
*Candida boidinii*	++	++	++

++, strong lytic effect; +, medium lytic effect; –, no lytic effect.

**Figure 5:**
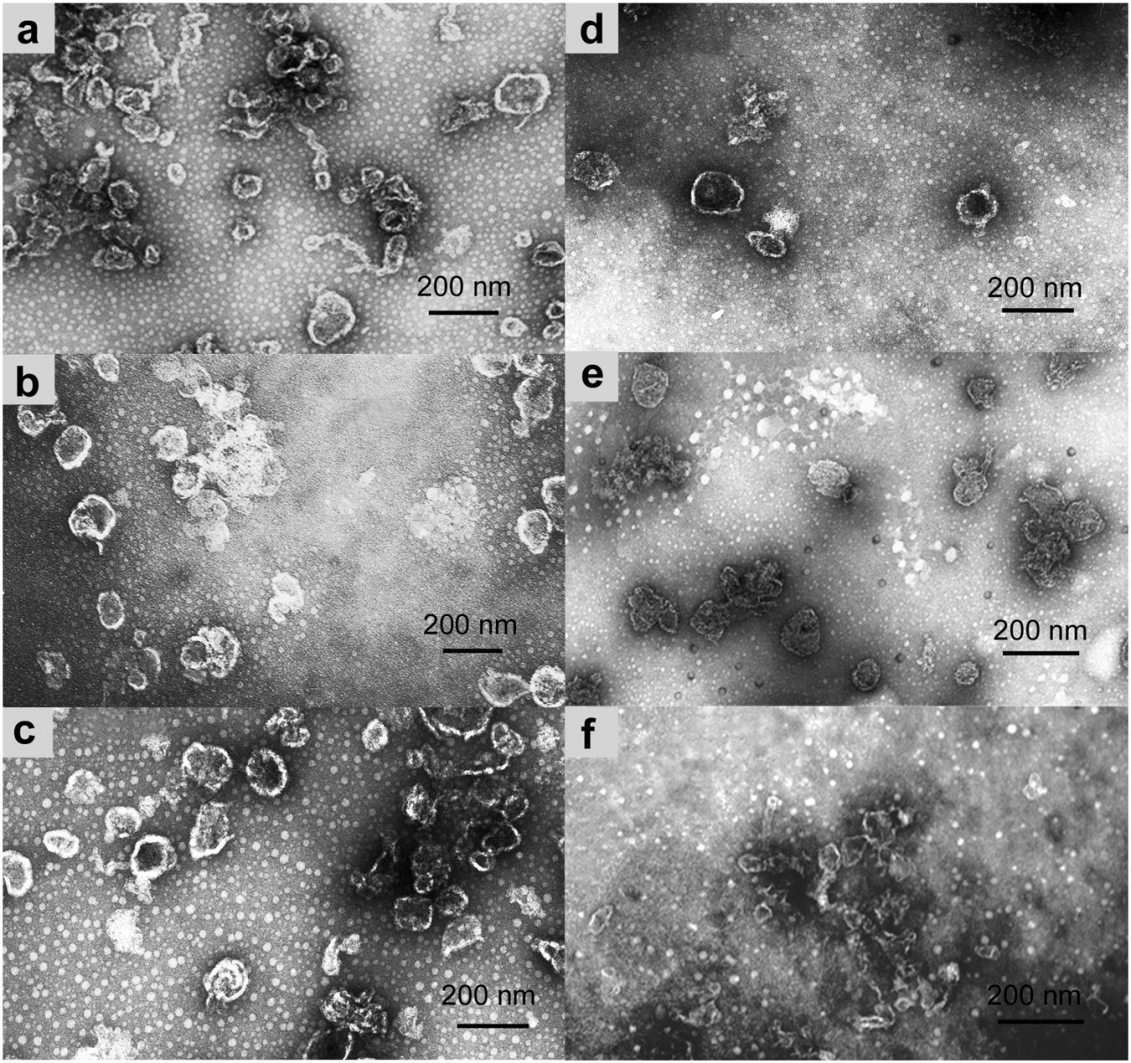
Negative staining electron microscopy of outer membrane vesicle preparations. (**a**–**c)** OMV fractions 8–10 (35% sucrose); (**d**–**f**) OMV fractions 11–13 (40% sucrose).

It is known that bacteria can form outer membrane vesicles that perform various functions. We suggested that *L. capsici* could form a subpopulation of OMVs containing an antifungal agent. To confirm this suggestion, the total preparation of OMVs was fractionated in a 30–55% sucrose density gradient. As the result, we obtained 22 fractions: 2–6, fractions distributed in a 30% sucrose; 7–10, fractions distributed in a 35% sucrose; 11–14, fractions distributed in a 40% sucrose; 15–18, fractions distributed in a 45% sucrose; 19–22, fractions distributed in a 50% sucrose. All fractions were analyzed by electron microscopy (Fig. 5).

The major part of OMVs was distributed between fractions 8–13. In fraction 8, OMVs were 82 ± 14 nm in diameter (Fig. 5a); fraction 9 contained the largest amount of OMVs 107 ± 16 nm in diameter as compared with the other fractions (Fig. 5b). Fractions 10 and 11 included OMVs of 111 ± 9 nm and 111 ± 8 nm in diameter (Fig. 5c,d); fraction 12, 96 ± 11 nm (Fig. 5e); and in fraction 13, only individual OMVs of 50 ± 11 nm in diameter on average occurred (Fig. 5f).

**Figure 6:**
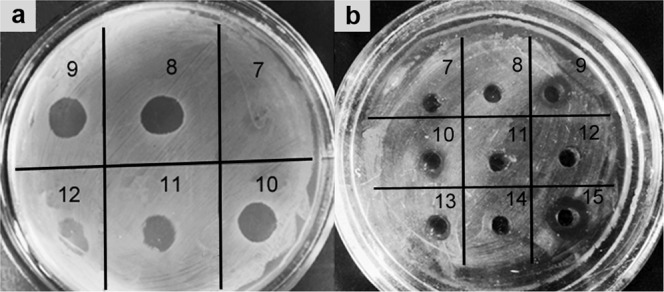
Lytic action of OMV fractions (7–12) after ultracentrifugation in the sucrose density gradient. (**a**), Antibacterial action with respect to *S. aureus* 209 P. (**b**), Antifungal action with respect to *Fusarium solani*: 13, culture liquid before vesicle precipitation; 14, culture liquid after vesicle precipitation; 15, initial vesicle preparation.

Comparative analysis of OMV lytic action in the obtained fractions showed fractions 8–13 to possess antibacterial activities (Fig. 6a). An antifungal activity was found predominantly in fraction 9 and insignificantly in fraction 10 (Fig. 6b).

Thus, an important result was obtained confirming that *L. capsici* cells formed a group of OMVs responsible for antifungal activity.

## Discussion

By the 1970s, the Canadian microbiologists Christensen and Cook proposed to single out from myxobacteria a group of microorganisms that differ by a number of properties. The main unifying feature by which this group was attributed to myxobacteria is their ability to lyse cells of prokaryotic and eukaryotic microorganisms. In a paper published in 1978, these investigators proposed a new genus *Lysobacter*. The novel genus then included four species: *L. enzymogenes* ATCC 29487 named so for its ability to produce two important proteolytic enzymes; *Lysobacter antibioticus* ATCC 29479, for its ability to produce the antibiotic myxin; *L. brunescens* ATCC 29482; *L. gummosus* ATCC 29489^1^. In the period from 1980 to 2000, the biochemistry of the *Lysobacter* bacteria had been dealt with by separate groups of investigators. Starting from the 2000s, research interest in these bacteria began to rise gradually, and in the recent five years the number of publications exceeded the previous years. A significant part of these publications has been research into the isolation of novel species; genetic studies have been published; to a smaller extent, biologically active substances have been described. It is evident to date that not all isolated species of *Lysobacter* possess expected antimicrobial activities^38–40^. Among relatively new species of this genus, *L. capsici* possesses an explicitly pronounced antimicrobial activity.

The species *L. capsici* YC5194^Т^ = VKM-2533^Т^ had been isolated in 2008 from the rhizosphere of pepper^41^. *L. capsici* produces lytic agents possessing an antimicrobial activity against plant pathogens, fungi and oomycetes^42^. Prior to our work, only the anti-nematode agents chitinase, gelatinase, anti-oomycete cyclic dipeptide cyclo(L-Pro-L-Tyr) and lactic acid had been isolated and characterized in *L. capsici*^29,32–34^.

It is known that the production of virtually all microbial substances strongly depends on storage and cultivation conditions. This is especially important for commercially valuable products of microbial synthesis. For *L. capsici*, the preservation of antimicrobial properties is complicated by the fact that the activity in question can be due to the combined effect of substances from different classes of compounds (proteins, antibiotics, peptides). Optimal conditions should be chosen for the preservation and efficient production of all these substances.

Earlier, we have shown that, to preserve the production of bacteriolytic enzymes in *Lysobacter* sp. XL1, the culture should be maintained on super-rich nutrient media^43^. In this work, we used the accumulated experience, and *L. capsici* was maintained on super-rich LBs, rich with PMA and cryopreserved. The maintenance of the culture in cryopreserved state was shown to be optimal for the preservation of the total lytic activity of *L. capsici*. This preserves the antifungal, antibacterial and yeast-lytic activities for the cultivation on various media. The storage of the culture on LBs leads to a virtually complete loss of antifungal activity. Herewith, the antibacterial activity is preserved at the cultivation on SYM, KSP and RM media. Storage on PMA leads to an almost complete loss of the antimicrobial properties on the whole.

If we analyze the component composition of the cultivation media, we can see that the concentration of protein hydrolysate (peptone, tryptone, casitone, aminopeptide) affects the exhibition of antibacterial activity. At a hydrolysate content of 10 g/L and higher this activity is observed to decrease. We should note here that this dependence is most probably determined by the production of bacteriolytic proteases. Thus, to preserve the production of these enzymes, it is important that the culture be maintained on super-rich media, and for the optimal revelation of their activity, the other way round, the cultivation on media with the low content of protein hydrolysate is required.

Also, we observe that at the maintenance and cultivation of *L. capsici* on rich media the antifungal activity is virtually lost. This activity could be suggested to be due to the production of an antibiotic. For this class of compounds it is known that on rich nutrient media their biosynthesis is not stable. For this reason, only cryopreservation is an optimal variant for the antibiotic activity to be retained. Herewith, a synthetic medium should be developed for the optimal manifestation of this activity.

Thus, *L. capsici* VKM B-2533^T^ is a potent producer of antimicrobial agents, which can include bacteriolytic enzymes.

A purification scheme was developed for isolation of *L. capsici* bacteriolytic enzymes. We succeeded in isolating four proteins in a homogeneous form and a mixture of two proteins (Table 2). According to MALDI-TOF, the isolated proteins comprise those identical to the α- and β-lytic proteases and the lysine-specific protease of *L. enzymogenes*. The serine proteases of MW 26 kDa and 29 kDa, and the protein identified as N-acetylglucosaminidase were isolated for the first time. Thus, these proteins are novel not only for the species *L. capsici* but also for the genus *Lysobacter* and require a detailed investigation.

Noteworthy among the isolated proteins is the β-lytic protease, which efficiently digests live cells of the methicillin-resistant staphylococcus. As a matter of fact, the β-lytic protease, along with the α-lytic protease, are the first bacteriolytic enzymes isolated in *Lysobacter*^37^. Both proteins have been characterized to various extents^44–47^. However, information about the recombinant β-lytic protease is absent in the literature. In this work, we succeeded in producing the recombinant protein in active state owing to the developed scheme of refolding. The produced expression system for the β-lytic protease based on *E. coli* BL21(DE3)/pLysE is of scientific value. Subsequently it can be used in studies of functionally significant sites for the antimicrobial activity of this protein to be revealed. At present, work is under way to develop a commercially valuable expression system for the β-lytic protease.

It is indisputable that bacteriolytic enzymes are promising for their use as the basis for new-generation antimicrobial agents. The problem of their active application is the complexity of developing biotechnologically valuable expression systems. Production of recombinant soluble bacteriolytic enzymes is problematic as it leads to the lysis of the producing strain. As the *Lysobacter* bacteria on the whole and the species *L. capsici* in particular can be considered to be a fount of such proteins, a solution of the problem can be to create homologous expression systems. *Lysobacter* genetic studies are now at an early stage of their development, so an efficient expression system for these proteins would hopefully appear in the near future.

As we already mentioned, the antimicrobial potential can be contributed to, besides enzymes and antibiotics, by outer membrane vesicles, which can contain all these agents. The vesicular subject area in Gram-negative bacteria was founded by the Americans Jagath L. Kadurugamuwa and Terry J. Beveridge, who carried out a comprehensive research into *Pseudomonas aeruginosa* vesicles. Those investigators laid the modern trends in vesicular studies, including the prospects of treating cancer and other diseases caused by pathogenic microorganisms^48–50^. At our laboratory, OMVs of *Lysobacter* sp. have been investigated for over ten years now^8,24,51^. Our research was pioneering in the field of OMV studies in *Lysobacter* bacteria. These days, one of the directions of our studies is the biogenesis of *Lysobacter* sp. XL1 OMVs and the role of the secreted bacteriolytic enzyme L5 in this process^51,52^.

This work established the ability to form outer membrane vesicles for *L. capsici* VKM B-2533^T^. The preparation of isolated OMVs possessed a potent antimicrobial action with respect to bacteria, fungi and yeasts. This activity can be due to both bacteriolytic enzymes and other antimicrobial agents. For instance, for *Lysobacter* sp. XL1 it has been shown that the bacteriolytic protease L5 gets into the medium by means of vesicles^8^. As a constituent of the vesicles, this enzyme lyses a broad range of bacteria, in contrast with its soluble form^53^. It proved noteworthy that all the antifungal activity of *L. capsici* concentrated in OMVs. A similar result has been obtained earlier for OMVs of *L. enzymogenes* C3^25^. The polycyclic tetramate macrolactam antibiotics have been identified in the vesicular preparation of this bacterium. It is known that vesicles can perform various functions in the bacterial cell^54,55^. We assume that particular groups responsible for various, including antifungal, functions, can be singled out in the population of OMVs produced by *L. capsici*. Indeed, during the separation of total OMVs in a sucrose density gradient we succeeded in producing a separate vesicular fraction that possessed an antifungal activity. Herewith, an antibacterial activity was found in all vesicular fractions. It can be assumed that different antimicrobial agents are responsible for antifungal and antibacterial activities. That said, there is a subpopulation of antifungal vesicles, too. This result is, in our view, very important for understanding the issues of vesicle biogenesis in Gram-negative bacteria. One of the possible biogenesis mechanisms is the involvement of the secreted product in this process^51,56^. It can be suggested that the antifungal agent of *L. capsici* affects the biogenesis of the OMV population by means of which it gets into the medium. This is an issue for further studies.

It is especially worth pointing out that the development of the *L. capsici* vesicular subject area has an evident biomedical trend. Vesicles that contain antimicrobial agents are a valuable model for the development of new-generation medicines on their basis^49,57^.

Thus, the research we did enabled important initial results for understanding the production of antimicrobial agents in *L. capsici*, for isolating bacteriolytic enzymes, including those not investigated earlier, for producing the recombinant β-lytic protease, for proving the occurrence of a group of outer membrane vesicles responsible for the antifungal activity. On the whole, the antimicrobial potential of *L. capsici* VKM B-2533^T^ can be characterized as significant and requiring thorough research.

## Materials and methods

### Strains and cultivation conditions

The work used strain *L. capsici* VKM В-2533^Т^.

The culture was maintained on agarized modified LBs (casitone, 33.0 g/L; yeast extract, 5.0 g/L; NaCl, 5.0 g/L, pH 7.2) and PMA (meat extract, 5.0 g/L; peptone, 10.0 g/L; NaCl, 5.0 g/L, pH 7.0) with reinoculations each ten days, as well as in cryopreservation.

For cultivation of *L. capsici*, we used liquid nutrient media of the following composition: KSP (casitone, 2.5 g/L; sucrose, 2.5 g/L; peptone, 2.5 g/L; KH_2_PO_4_, 0.1 g/L; K_2_HPO_4_, 0.1 g/L; MgSO_4_×7H_2_O, 0.1 g/L, pH 7.2);^58^ RM (glucose, 5.0 g/L; peptone, 2.0 g/L; yeast extract, 2.0 g/L; Na_2_HPO_4_×12H_2_O, 4.2 g/L; KH_2_PO_4_, 1.0 g/L; KCl, 0.6 g/L; MgSO_4_×7H_2_O, 5.0 g/L, pH 7.0);^59^ modified LB (peptone, 10.0 g/L; yeast extract, 5.0 g/L; NaCl, 5.0 g/L, pH 7.0); PYKM (peptone, 10.0 g/L; yeast extract, 5.0 g/L; KH_2_PO_4_, 1.4 g/L; MgSO_4_×7H_2_O, 1.0 g/L, pH 7.0);^60^ SYM (sucrose, 10.0 g/L; yeast extract, 5.0 g/L, pH 7.0)^60^, medium 5/5 developed at the IBPM RAS (yeast extract, 1.0 g/L; soybean extract, 30.0 g/L; tryptone, 5.0 g/L; aminopeptide, 60.0 g/L, pH 7.2). The cultivation was run at 29°С with aeration for 20 h, which corresponds to the stationary culture growth phase and the maximal bacteriolytic activity.

As test objects to study the lytic potential of L. capsici, we used the bacteria Bacillus subtilis W-23, B. cereus 217, Staphylococcus aureus 209 P, S. aureus 55 MRSA, Micrococcus roseus В 1236, M. luteus В 1819, Proteus vulgaris H-19, Pseudomonas fluorescens 1472, Escherichia coli K12; the mycelial fungi Fusarium solani, Sclerotinia sclerotiorum, Aspergillus niger; and the yeasts Candida boidinii, C. utilis. Bacterial test cultures were grown on medium 5/5; yeasts and mycelial fungi, on wort agar. Agarized nutrient media contained agar at a concentration of 1.5%.

Recombinant strains *Escherichia coli* BL21(DE3)/pLysE were grown on LB medium containing 0.03 mg/mL chloramphenicol and 0.10 mg/mL ampicillin at 37°С.

### Spot assay of lytic action

The lytic action of preparations was determined by the spot test of live bacteria. A preparation in the amount of 10 μL was applied onto a grown lawn of bacterial target cells. Dishes were dried for 30 min and incubated at 29 °C overnight. In the case of live mycelial fungi, 35 μL of the preparation each was introduced into wells made in a medium with a lawn of target cells. Dishes were incubated for 48 h at room temperature. The lytic action on test cultures was indicated by the appearance of lysis zones in sample application spots. A strong lytic effect manifested itself as a clearcut visible area of lysis in the application spot. A weak lytic action showed as a weakly transparent lysis area in the application spot. Sterile 10 mM Tris-HCl, pH 8.0, was used as a control.

### Isolation of bacteriolytic enzymes

The culture of *L. capsici* cells was grown for 20 h with aeration. Cells were discarded by centrifugation at 5000*×g* and 4°С for 20 min. Culture liquid proteins were fractionated by ammonium sulphate stepwise at 0–60% and 60–80% saturation. Residues of the fractions were produced by centrifugation at 25 960*×g* and 4°С for 60 min. The residue of the fraction at 60–80% saturation was dissolved in 50 mM Tris-HCl, pH 8.0, and dialyzed using a dialysis tubing of 10 kDa MW cut-off against the same buffer at 4°С overnight.

The dialysis was followed by cation exchange chromatography on a Toyopearl CM-650М column (Tosoh, Japan) equilibrated with 50 mM Tris-HCl, pH 8.0. After washing the column, isocratic elution with 0.3 M NaCl in the same buffer was done. Lytically active elution fractions were combined and dialyzed against 50 mM Tris-HCl, pH 8.0. As the wash-out had a lytic activity, it was also dialyzed against the same buffer. The subsequent stages of purifying elution and wash-out enzymes were the same. After the dialysis, cation exchange chromatography on an ENrich S column (Bio-Rad, USA) equilibrated with the same buffer was conducted again, using an NGC chromatographic system (Bio-Rad, USA). After washing the column, elution in a NaCl linear gradient from 0 to 1 M was done. Lytically active fractions were combined and subjected to gel filtration using the same chromatographic system and a HiLoad 16/60 (Superdex 75) column (Amersham Biosciences, Sweden) equilibrated with 50 mM Tris-HCl, 0.5 M NaCl, pH 8.0.

### MALDI-TOF mass spectrometry

Protein bands were excised from acrylamide gels after electrophoresis under denaturing conditions (SDS-PAGE), washed in 50% (v/v) acetonitrile, 50% (v/v) 50-mM ammonium bicarbonate at 37 °C and dehydrated in acetonitrile. After that, a 10-μl trypsin solution (20 μg/mL) in 50-mM bicarbonate buffer was added (sequencing grade, Promega USA), and the mixture was incubated for 16 h at 37 °C. The hydrolysate in the amount of 0.5 μL was mixed on a mass spectrometer (Bruker, Germany) target with an equal volume of a solution of 2,5-dihydroxybenzoic acid in 50% acetonitrile and 3% TFA, and air-dried.

The mass spectra of trypsin-digested proteins were acquired using a MALDI-TOF/TOF mass spectrometer (Ultraflex, Bruker Daltonics, Germany) equipped with an Nd:YAG laser in the reflector mode. Monoisotopic [MH^+^] ions were measured in the *m*/*z* range of 700–3500 with a tolerance of 50 ppm. Fragment ion spectra were obtained in the LIFT mode. The accuracy of fragment ion mass peak measurements was within 1 Da^61^.

The proteins were identified using the MASCOT search software (peptide fingerprinting combined with the ion search option; www.matrixscience.com^62^. The search was carried out using the NCBI.nr database with no taxonomy limits. Candidate proteins were considered as reliably identified when the score was greater than 83 (*p* < 0.05). For the search of candidate proteins in combined ms + (ms-ms) data, Biotools 3.0 (Bruker Daltonics) was used^61^. Homologues of isolated proteins were searched for using the BLAST databases (https://blast.ncbi.nlm.nih.gov)^63^.

### Bacteriolytic activity assay

The bacteriolytic activities of the preparations were determined by turbidimetry. As substrates, autoclaved or live *S. aureus* 209 P cells were used. Cells of the staphylococcus were suspended in 10 mM Tris-HCl, pH 8.0, to an optical density of OD_540_ = 0.5. An enzyme preparation in the amount of 50 μL was added to a 0.950-mL substrate, and the mixture was incubated at 37 °C for 5 min. The reaction was arrested by placing test tubes on ice; the absorption of the suspension was measured at 540 nm. An amount of enzyme leading to a decrease in the absorption of the cell suspension by 0.01 optical units at 37 °C per 1 min was taken as a unit of bacteriolytic activity (LU).

### Minimal inhibitory concentration assay

Enzyme preparations at final concentrations of 0.19, 0.48, 0.95, 1.90, 2.85, 3.80 and 4.75 μg/mL were added to 1-mL suspensions of *S. aureus* 55 MRSA cells of OD 0.5. The mixtures were held for 48 h. After that, 100-μL suspensions were plated onto agarized nutrient media and cultivated at 37 °C for 18 h. The MICs of the enzymes were determined by live staphylococcal cell count.

### Production of OMVs

The preparation of outer membrane vesicles was produced by differential centrifugation from the culture liquid of *L. capsici* grown on RM medium. Cells from a 0.3-L culture were discarded by centrifugation at 7 500*×g* for 20 min at 4 °C. OMVs were precipitated from the produced culture liquid by centrifugation at 113 000*×g* for 2 h at 4 °C. The OMV residue was washed with 50 mM Tris-HCl, pH 8.0, by centrifugation at the same speed for 1 h. The produced OMV residue was resuspended in equal volumes of 50 mM Tris-HCl, pH 8.0, and stored at –20 °C.

### Fractionation of OMVs in sucrose density gradient

The preparation of *L. capsici* OMVs was fractionated in a sucrose density gradient according to an earlier developed protocol. The OMV residue was washed with 50 mM Tris-HCl, pH 8.0, followed by centrifugation at the same speed; the residue was then dissolved in 2 mL of 25% sucrose in 10 mM Tris-HCl, 5 mM EDTA, pH 8.0. The OMV preparation thus produced was layered on a stepwise sucrose density gradient (30–55% sucrose in 10 mM Tris-HCl, 5 mM EDTA, pH 8.0) at a step of 5% sucrose. Centrifugation was run at 106 500*×g* for 12 h. Fractions were collected in equal volumes of 500 μL, starting from the meniscus. As the result, 22 OMV fractions were taken. The fractions were diluted to a volume of 30 mL with 10 mM Tris-HCl pH 8.0 and centrifuged at 115 000*×g* for 2 h. The residues were resuspended in equal volumes of 50 mM Tris-HCl, pH 8.0^51^.

### Cloning and expression of *L. capsici* VKM B-2533^T^ β-lytic protease

The β-lytic protease gene (*βl*) was amplified with the chromosomal DNA isolated using a Genomic DNA Purification Kit (Thermo Scientific, USA) according to the manufacturer’s recommendations. To amplify the target sequence (NCBI reference sequence of protein: ALN84242.1), oligonucleotides were designed in OligoAnalizer3.1 (http://eu.idtdna.com/calc/analyzer) using the bioinformatics data of the *L. capsici* 55 genome in the GenBank. The signal peptide was searched for in SignalP 4.1 package (http://www.cbs.dtu.dk/services/SignalP/). Oligonucleotides (Table 4) were synthesized by Evrogen (Moscow, Russia). Oligonucleotides containing restriction endonuclease recognition sites were used for cloning into the vector pET19(mod)^64^. The target gene was amplified on a Mastercycler ProS (Eppendorf, Germany). The protocol for the PCR with the DNA-dependent DNA polymerase Q5 (New England Biolabs, USA) was as follows: primary heating, 98°С for 30 s; 35 cycles: 98°С for 10 s, 60°С for 20 s, 72°С for 45 s; additional elongation for 2 min in the last cycle.

**Table 4:** Oligonucleotides used in this study.

Oligonucleotides used for cloning		
Forward primer	*L. capsici* genomic DNA	5’-TATATCATATGGCAGAGCGCGGCGGGTTG-3’
Reverse primer	*L. capsici* genomic DNA	5’- TATATGGATCCTCAGTTCGGGCCTGGGTTG-3’
**Oligonucleotides used for sequencing**		
Forward primer	Plasmid pET19(mod)	5’- GAATGTAGGGGGAACAGCGC-3’
Reverse primer	Plasmid pET19(mod)	5’-CCCCTCAAGACCCGTTTAGAG-3’

The restriction treatment and DNA ligation were carried out according to the standard techniques described in ref. ^65^. The restriction treatment was done using FastDigest NdeI and FastDigest BamHI (Thermo Fisher Scientific, USA). The T4 DNA Ligase (Thermo Fisher Scientific, USA) was used for ligation.

Insertion into the expression vector was verified by the PCR using oligonucleotides to the β-lytic protease gene followed by electrophoresis in 0.8% agarose gel with addition of ethidium bromide. The electrophoresis parameters were set to *I* = 100 mA, *U* = 100 V, *P* = 15 W. To determine the lengths of separated DNA fragments, we used SM0331 GeneRuler DNA Ladder Mix (Thermo Fisher Scientific, USA): 100, 200, 300, 400, 500, 600, 700, 800, 900, 1000, 1200, 1500, 2000, 2500, 3000, 3500, 4000, 5000, 6000, 8000, 1000 bp. The plasmid DNA was isolated using a Quantum PrepPlasmid Miniprep Kit (Bio-Rad, USA) according to the manufacturer’s recommendations.

The recombinant plasmid pET19(mod)-Bl was transformed into chemically competent *E. coli* BL21(DE3)/pLysE cells produced according to the protocol in ref. ^66^. The cultures were grown on an antibiotics-containing LB medium to OD_540_ = 0.7, were induced with 1 mM IPTG, then the cultivation was continued at 37 °C for 4 h.

To establish the complete nucleotide sequence of the gene *βl*, this gene with the genomic DNA flanking sequence (the N-end of the *βl* gene product was flanking) was cloned into the plasmid pET19(mod). The oligonucleotides were designed (Table 4).

The sequences of all cloned DNA fragments were confirmed by sequencing at Evrogen (Moscow).

### Purification of recombinant β-lytic protease

*E. coli* BL21(DE3)/pLysE was used as an expression system for the β-lytic protease. The target protein accumulated in cells of the recombinant strain as inclusion bodies. The bodies were produced by ultrasonic disruption of cells (mode, medium; amplitude, 4; 15 s; pause, 15 s; repeated 6 times) on an ice bath. The pro-protein was purified on a His Trap FF 5 mL (GE Healthcare, USA) using an NGC system (Bio-Rad, USA). The column was equilibrated with 50 mM Tris-HCl, 8 M urea, 1 M NaCl, 20 mM imidazole, pH 7.8. The fraction containing the target protein was dialyzed against 50 mM Tris-HCl, 8 M urea, 5 mM 2-mercaptoethanol, pH 8.0.

### Development of renaturation conditions for β-lytic protease

The following buffers were chosen for renaturation: buffer 1–50 mM Tris-HCl, 0.5 M arginine, 0.05% Chaps, pH 8.5; buffer 2–50 mM Tris-HCl, 0.5 М arginine, 5 mM cysteine, 1 мМ cystine, 0.05% Chaps, pH 8.5; buffer 3–50 mM Tris-HCl, 0.5 М arginine, 0.25 М NaCl, 0.40 М sucrose, 5 mM 2-mercaptoethanol, 0.05% Chaps, pH 8.5; buffer 4–0.8 М Tris-HCl, 5 mM cysteine, 1 мМ cystine, 0.05% Chaps, pH 8.5; buffer 5–0.8 М tricine, 5 mM GSH, 1 mM GSSG, 0.05% Chaps, pH 8.5. The enzyme preparation was dialyzed against the chosen buffers for 44 h without mixing at 7 °C. After that, aggregated proteins were discarded by centrifugation at 10 000*×g* for 20 min. Then the dialysis was repeated against 50 mM Tris, pH 8.0. Newly formed aggregated protein molecules were discarded by centrifugation under the same conditions. The produced samples were incubated at room temperature for 44 h. The efficiency of renaturation was assessed by electrophoresis under denaturing conditions and by measurement of the bacteriolytic activity with respect to autoclaved and live cells of *S. aureus* 209 P. Purification of the β-lytic protease recombinant protein to an electrophoretically homogeneous state was done using an ENrich S column (Bio-Rad, USA) and an NGC chromatographic system (Bio-Rad, USA).

### Electrophoresis of proteins in polyacrylamide gel

For the electrophoretic assay, proteins from the analyzed preparations were sedimented with trichloroacetic acid at a final concentration of 10% or were used without sedimentation in the case of their subsequent staining with zinc^67^. Protein residues were analyzed by electrophoresis^68^ with 0.1% SDS in 12.5% PAG. Electrophoresis in stacking gel was run at 90 V; in separating gel, at 180 V. Protein bands in gels were revealed by staining with a solution of Coomassie Brilliant Blue R-250 (Serva, Germany) and imidazole–ZnCl_2_ solutions. As molecular weight markers, we used SM0431 (Thermo Fisher Scientific, USA): β-galactosidase (116 kDa), bovine serum albumin (66.2 kDa), ovalbumin (45 kDa), lactate dehydrogenase (35 kDa), restrictase Bsp981 (25 kDa), β-lactoglobulin (18.4 kDa) and lysozyme (14.4 kDa).

### Protein concentration assay

Protein concentrations in preparations were determined by the Bradford protein assay^69^. The reactions were conducted according to the manufacturer’s protocol for the Coomassie reagent (Thermo Scientific, USA). The protein concentration was determined by the calibration curve plotted for an aqueous solution of BSA (Sigma, USA) within the range of 1–25 μg/mL.

### Transmission electron microscopy

Samples of OMVs were examined by transmission electron microscopy. The samples were placed on formvar-coated copper grids and allowed to stay for 2 min. The sample excess was removed using filter paper. After drying, the samples were treated with a 0.3% aqueous solution of uranyl acetate (pH 4.0) for negative staining. Negatively stained preparations were examined with a JEOL 1200EX transmission electron microscope (Akishima, Japan) at an accelerating voltage of 80 kV^52^.

### Ethical statement

The present study does not contain any experiments in relation to either human participants or animal models by any of the authors.

## Supplemenatry information

Supplemenatry information.
