# Supplementary material for: Lytic potential of Lysobacter capsici VKM B-2533T: bacteriolytic enzymes and outer membrane vesicles
Source: Sci Rep. 2020 Jun 19;10:9944. doi: 10.1038/s41598-020-67122-2 (PMC7305183; doi:10.1038/s41598-020-67122-2)
Supplement: Supplementary file 1 — Supplemenatry information. [file 41598_2020_67122_MOESM1_ESM.pdf]

Lytic potential of *Lysobacter capsici* VKM B-2533^{T}: bacteriolytic enzymes and outer membrane vesicles

A.S. Afoshin[1], I.V. Kudryakova[1], A.O. Borovikova[1], N.E. Suzina[1], I.Yu. Toropygin[2], N.A. Shishkova[3], N.V. Vasilyeva[1,*]

[1] Laboratory of Microbial Cell Surface Biochemistry, G.K. Skryabin Institute of Biochemistry and Physiology of Microorganisms, Russian Academy of Sciences, PSCBR RAS, 5 Prosp. Nauki, Pushchino, Moscow Region, 142290 Russia

[2] Department of Proteomics, V.N. Orekhovich Research Institute of Biomedical Chemistry, Russian Academy of Medical Sciences, 10 Pogodinskaja Str., Moscow, 119832, Russia

[3] Laboratory of Anthrax Microbiology, FBIS State Research Center for Applied Microbiology and Biotechnology, Obolensk, Serpukhov District, Moscow Region, 142279, Russia

***Corresponding author**:
N.V. Vasilyeva, 5 Prosp. Nauki, Pushchino, Moscow Region, 142290, Russia
Tel: +7(495)625-74-48
Fax: +7(495)956-33-70
E-mail address: vasilyevanv@rambler.ru

Supplementary Table S1 Purification of *L. capsici* bacteriolytic proteins.

Step	Total protein (mg)	Total activity (U)	Specific activity (U/mg)	Purification (fold)	Yield (%)
Culture liquid	11.57	216 720	18 731	1.00	100.0
CM 650 elution fractions					
CM 650 elution fractions	3.24	54 000	16 667	0.890	24.92
ENrich S	2.33	36 000	15 451	0.825	16.61
Superdex 75 α-Lytic protease	0.33	4 290	13 000	0.694	1.98
Superdex 75 β-Lytic protease	0.92	27 917	30 345	1.620	12.88
Superdex 75 Serine trypsin-like proteases	0.12	884	7 367	0.393	0.41
CM 650 washing fractions					
CM 650 washing fractions	7.21	41 480	5753	0.307	19.14
ENrich S N-acetylglucosaminidase	0.002	0.11	55	0.003	5.08 10^{-5}
Superdex 75 Serine protease	0.179	107	598	0.032	0.05

As *L. capsici* produces a large amount of lytic agents that have additive effects on the total bacteriolytic activity of the culture liquid, a change in the degree of purification in the course of the process is not typical of all bacteriolytic enzymes. A consequence of this is a decrease in the specific activity of the target protein at an increase of the degree of its purification.

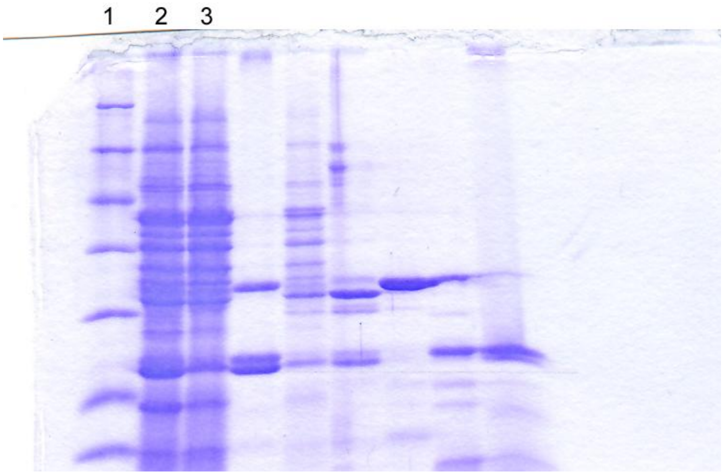

Supplementary Figure 1 Original gel images for Fig. 2b: lanes 1, 2, 3 correspond to lanes M, 1, 2 of Figure 2b.

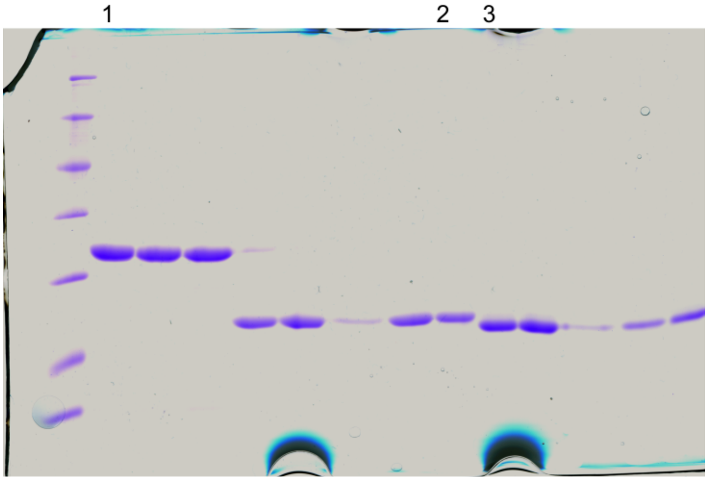

Supplementary Figure 2 Original gel images for Fig. 2b: lanes 1, 2, 3 correspond to lanes 3, 4, 5 of Figure 2b.

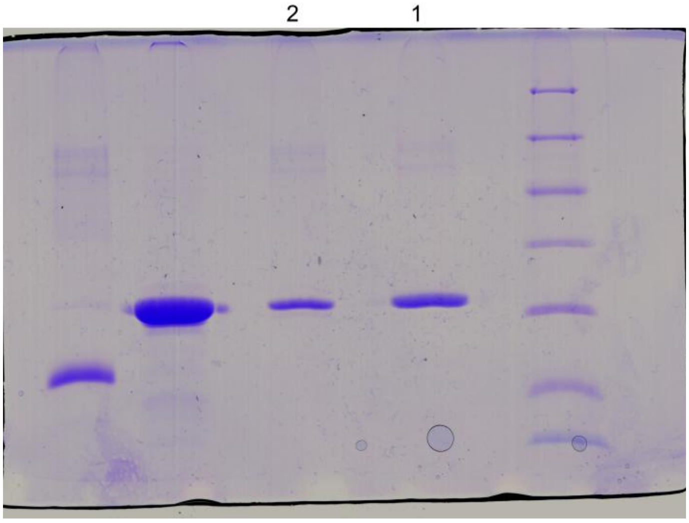

Supplementary Figure 3 Original gel images for Fig. 2b: lanes 1, 2 correspond to lanes 6, 7 of Figure 2b.

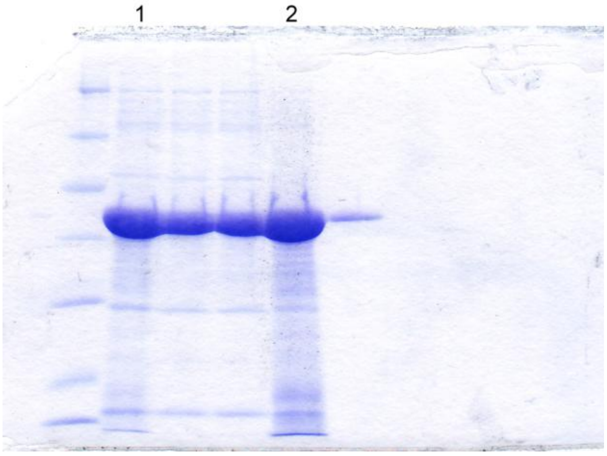

Supplementary Figure 4 Original gel images for Fig. 3a: lanes 1, 2 correspond to lanes 1, 6 of Figure 3a.

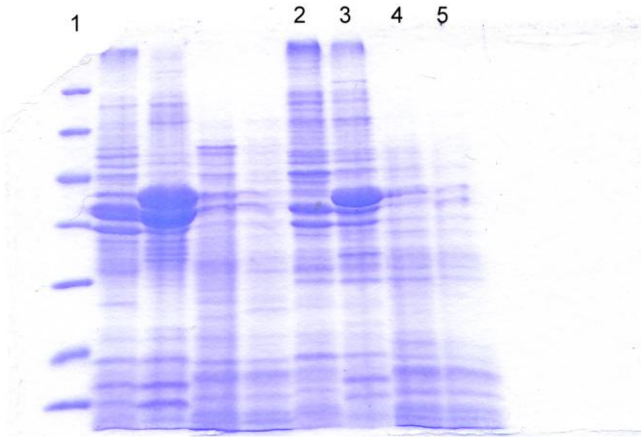

Supplementary Figure 5 Original gel images for Fig. 3a: lanes 1, 2, 3, 4, 5 correspond to lanes M, 2, 3, 4, 5 of Figure 3a.

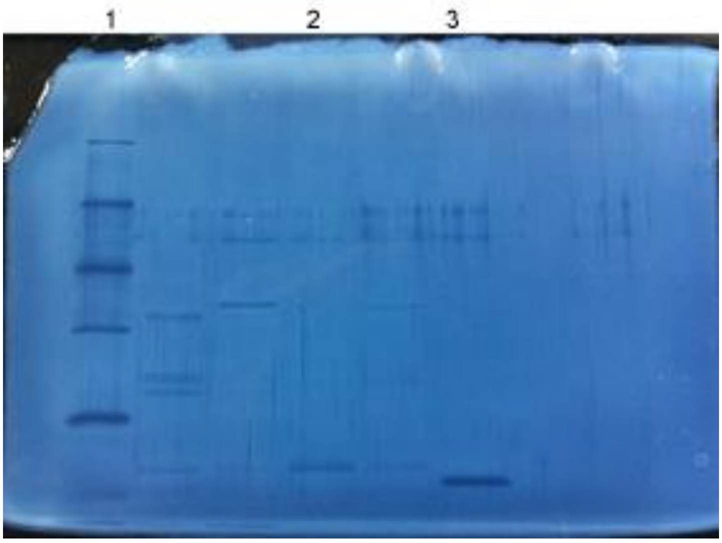

Supplementary Figure 6 Original gel images for Fig. 3c: lanes 1, 2, 3 correspond to lanes M, 1, 2 of Figure 3c.

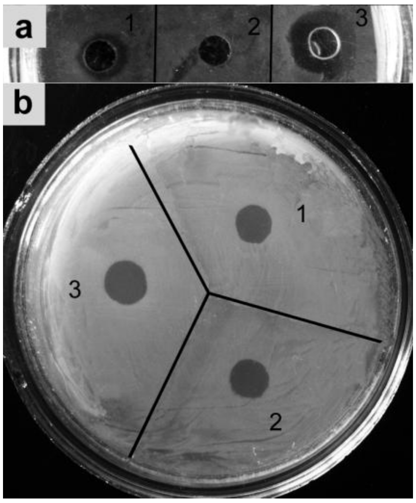

Supplementary Figure 7 Lytic action of OMV culture liquid. **a**, target cells of *S. sclerotiorum*. **b**,target cells of *M. luteus*. Culture liquid before (1) and after (2) isolation of OMVs, and preparation of OMVs (3).
